# Anti-HEV IgG Avidity Testing: Utility for Diagnosing Acute and Resolved Genotype 3 Infections

**DOI:** 10.3390/v13020236

**Published:** 2021-02-03

**Authors:** Claudia Minosse, Daniele Lapa, Antonio Coppola, Federica Rapagna, Gianpiero D’Offizi, Chiara Taibi, Raffaella Lionetti, Maria Rosaria Capobianchi, Fiona McPhee, Anna Rosa Garbuglia

**Affiliations:** 1 Laboratory of Virology, “Lazzaro Spallanzani” National Institute for Infectious Diseases, IRCCS, 00149 Rome, Italy; claudia.minosse@inmi.it (C.M.); daniele.lapa@inmi.it (D.L.); antonio.coppola@inmi.it (A.C.); maria.capobianchi@inmi.it (M.R.C.); 2 Eurospital SpA, Via Flavia 122, 34147 Trieste, Italy; frapagna@eurospital.it; 3 Hepatology and Infectious Diseases Unit, “Lazzaro Spallanzani” National Institute for Infectious Diseases, IRCCS, 00149 Rome, Italy; gianpiero.doffizi@inmi.it (G.D.); chiara.taibi@inmi.it (C.T.); raffaella.lionetti@inmi.it (R.L.); 4 Bristol-Myers Squibb, Cambridge, MA 02142, USA; fiona.mcphee@bms.com

**Keywords:** hepatitis E virus, IgG avidity test, IgM, HEV RNA, HEV genotype 3, HEV acute hepatitis

## Abstract

European Association of the Study of the Liver (EASL) guidelines specify HEV RNA, as well as anti-HEV IgG and IgM as positive markers for acute HEV infection. HEV RNA assay sensitivity limitations may lead to false negative test results in patients with low levels of viremia. Moreover, anti-HEV IgM positivity is not a reliable indicator for distinguishing between acute and resolved infections given the ability of this antibody to persist several months after a resolved infection. Our study aims were to assess HEV IgG avidity for diagnosing acute and resolved infections, regardless of the anti-HEV IgM serostatus, and examine assay reliability when evaluating different genotype 3 (GT3) HEV subtypes. Patient serum samples (*n* = 104) were tested for HEV IgG avidity by utilizing the DIA.PRO kit on a DSX automated instrument. Among patients identified with acute HEV infections, 32 were infected with GT3: GT3c (*n* = 5), GT3e (*n* = 8), 3f (*n* = 17) and GT3-unsubtyped (*n* = 2). Avidity sensitivity was 91.2% and specificity was 100%. For patients with long-lasting anti-HEV IgM persistence, an Avidity Index >70% was observed. Thus, the DIA.PRO avidity assay may be utilized to distinguish between recently acquired and resolved HEV GT3 infections. However, for equivocal results (Avidity Index > 40–70%), HEV RNA molecular testing will be required to confirm a recent infection.

## 1. Introduction

Hepatitis E virus (HEV) is the main cause of acute viral hepatitis globally. It is estimated that there are approximately 20 million new infections every year with a worldwide yearly mortality rate of almost 70,000 people [1,2,3]. HEV is a single-stranded, positive sense RNA genome virus with a non-enveloped capsid, belonging to the genus *Orthohepevirus* of the *Hepeviridae* family. In general, human infections are linked to *Orthohepevirus* species A, which comprises eight genotypes [4]. Genotypes 1 and 2 are restricted to humans, while genotype 3 (GT3) and 4 (GT4) are considered zoonotic, causing cross-species infection in, for example, pigs, wild boars, deer, rabbits and rats. GT3 and GT4 are frequently transmitted to humans via contaminated food. HEV GT3 is predominantly found in Western countries [5] while GT4 mainly circulates in Asia [6], although some sporadic cases have also been identified in European countries [7,8]. Of the reported GT3 subtypes, GT3c, GT3e and GT3f have been isolated from European human samples [5,9,10,11]. GT7 and GT8 infect dromedary and Bactrian camels, respectively, with sporadic transmission to humans being reported for GT7 [12,13]. Recently, another HEV species, HEV-C (*Orthohepevirus* species C), which is generally isolated from rodents and ferrets, has also been detected in humans [14,15].

Individuals with acute HEV infection can be symptomatic or asymptomatic. Reported symptoms are indistinguishable among the HEV genotypes and are very similar to those observed for hepatitis A virus acute infections. Symptoms can be non-specific (the prodromal or pre-icteric phase of an acute infection) and include asthenia, fever and pain. This may be followed by an icteric phase of morphologic changes (hepatocyte cytolysis) and biochemical changes in alanine amino transferase (ALT), aspartate aminotransferase (AST) or bilirubin [16]. Progression to a fulminant hepatitis has been observed in infected individuals with underlying liver disorders. Additionally, immunocompromised patients infected with HEV GT3 have developed chronic hepatitis [17].

A chronic infection is characterized by the persistence of HEV RNA viremia for more than six months after diagnosis [18]. While EASL guidelines indicate the detection of HEV RNA alone or with anti-HEV IgG and/or anti-HEV IgM as parameters for HEV acute infections [17], ECDC suggests that anti-HEV IgG plus IgM positivity is an indispensable indicator for HEV acute hepatitis. HEV RNA is the first indicator of infection and it remains detectable for approximately 10 to 52 days in serum [19]. However, when HEV RNA levels are <100 IU/mL, false negative test results are possible [19]. Moreover, the instability of naked HEV RNA may also influence assay sensitivity [20], in addition to sub-optimal storage and freeze/thaw techniques, as observed by conventional amplification methods (RT-PCR) [21] of other viral samples. Anti-HEV IgM can be detected within three to four days after the onset of jaundice [22] and persist up to 225 days after the onset of reported symptoms [23]. Detection of anti-HEV IgG is possible three to four days after anti-HEV IgM and persists for at least seven years. Anti-HEV IgG titre is variable and depends on the assay used for detection and thus, positivity is not useful for distinguishing between acute and resolved infections. IgG avidity is a quantitative serological test used to discriminate recent and resolved infections. IgG antibody binding power is weak in the early stage of infection, while it increases progressively in subsequent weeks due to affinity maturation and antigen-driven B cell selection. Avidity assays have been employed to differentiate acute or primary infections from persistent infections, recurrent infections, or the reactivated disease [24,25,26]. With respect to HEV infection, some studies have reported the role of anti-HEV IgG avidity in the improvement of diagnosis of acute infections [27,28]. However, this benefit requires further investigation in the context of different HEV subtypes and potential cross-reactivity. Our study aims to assess whether differentiation between acute and resolved HEV infections is possible by using the anti-HEV IgG avidity test, as well as to establish whether the reliability of the test is influenced by the GT3 subtype.

## 2. Materials and Methods

### 2.1. Study Population

Serum samples (*n* = 104) were collected from 101 patients and analysed. These samples were either obtained from patients recruited to the INMI L Spallanzani Hospital IRCCS or shipped to the virology lab for HEV RNA and antibody testing in a blinded fashion. This research was approved by the local Ethical Committee (n.70/2018). Sample data were stratified according to HEV disease status, serology and RT-PCR results. Group A (*n* = 34) included sera from patients with a confirmed acute infection, as determined by positive results for HEV RNA and anti-HEV IgG and IgM. The HEV genotype and subtype status was evaluated for these patients. Group B (*n* = 16) included patients who had increases in transaminases, liver disorders, pain, or asthenia in the months prior to virological testing. At the time of sample collection, these patients had recovered from acute HEV infection, did not demonstrate chronic symptoms and exhibited decreases in transaminase levels. Testing indicated that these patients were HEV RNA negative and anti-HEV IgG and IgM positive (see Table 1). Group C (*n* = 15) included patients who had been HEV RNA positive and recovering from acute infection. At the follow-up visit, collected samples were HEV RNA negative but positive for both anti-HEV IgG and IgM and liver function tests were now normal. These follow-up samples were collected 1 month to 2 years after the initial positive HEV RNA diagnosis (see Table 1). Group D included patients with resolved HEV infection (*n* = 5), as determined by anti-HEV IgG positive and IgM negative results. One patient (Pt71) was HEV RNA positive, although anti-HEV IgG and IgM were not detected. This patient was included in Group D to check the avidity test reliability in viremic HEV patients with acute infection, who has not already developed either anti-HEV IgG or anti-HEV IgM.

In order to investigate potential cross-reactivity of the DIA.PRO anti-HEV avidity assay, patient samples infected with hepatotropic viruses other than HEV were also evaluated. One patient (Pt72) was HEV RNA negative, anti-HEV IgG and IgM positive, HBV DNA positive and anti-HBc IgM positive. An acute hepatitis A (HAV) infection was confirmed in 12 patients (Pts 73–84) by HAV RNA RT-PCR testing, while an acute Epstein-Barr virus (EBV) infection was diagnosed in 10 patients (Pts 85–94) with the presence of EBV DNA and anti-virus capsid antigen (VCA) IgM in their blood. Ten patients (Pts 95–104) were CMV DNA positive and anti-cytomegalovirus (CMV) IgM positive. All these samples (Pts 73–104) were HEV RNA negative.

Detailed sample information is described in Table 1.

### 2.2. Avidity Test

The avidity test is based on the detection of dissociation of previously formed HEV IgG antigen immunocomplexes through a dissociation complex agent (urea) treatment. IgG antibodies bind weakly to the antigen during the initial stage of infection. After a month, this interaction strengthens and avidity increases progressively. Results are expressed as Avidity Index (AI), which is calculated as follows: AI = OD_450_ (anti-HEV IgG in the urea-treated well)/OD_450_ (anti-HEV IgG in the untreated well) × 100 where OD_450_ is the optical density at a wavelength of 450.

In this study, the avidity test was performed using the DIA.PRO HEV IgG assay, which is an enzyme-linked immunosorbent assay based on four synthetic peptides with conservative epitopes in ORF2 and ORF3 from genotypes 1, 2, 3, and 4 (DIA.PRO, Milan, Italy).

Serum samples were diluted in order to have an OD_450_ in the range of 0.460 to 3.3. The assay was performed using the automated DSX Elisa processing system. Fresh tips were used to respectively transfer samples and reagents. This automated system allowed minimal hand-on time and yielded a result in 3 h 40 min. The total assay volume was 200 µL (pre-diluted 1:20). The liquid levels of samples, reagents, starter reagents, and system fluid were checked via sensors.

Duplicate samples were processed using DIA.PRO anti-HEV IgG enzyme-linked immunosorbent reagents.

One sample was prepared following standard procedures while the other was initially washed three times with 300 µL urea (6M final concentration in washing buffer), which also included a 37 °C soak for 5 min between washes. The assay was then performed according to the manufacturer’s recommendations for both duplicates. The assay was repeated three times.

Results were categorized as follows: low Avidity Index, 1–40%; equivocal results, >40–70%; high Avidity Index: >70%.

To confirm long-lasting anti-HEV IgM positivity, samples were evaluated using the “One site HEV IgM rapid test” (CTK Biotech, Poway, CA, USA).

### 2.3. Data Analysis

Assay sensitivity was calculated for Group A samples as follows: true positive/(true positive + false negative) × 100). Assay specificity was calculated in Group D (Pts73–104) samples as follows: true negative/(true negative + false positive) × 100.

The comparison of AI in different GT3 subtypes was performed using the Mann–Whitney test with GraphPad Prism version 8.0.2 (GraphPad software, San Diego Inc., CA, USA). A *p*-value < 0.05 was considered statistically significant.

The Wald test was employed to calculate the kappa value and was performed using the Analyse-it software, version 5.65.3. A kappa value > 0.70 indicated high agreement, 0.40 to 0.70 indicated moderate (or good) agreement and <0.40 indicated poor agreement.

### 2.4. Phylogenetic Analysis

HEV RNA was isolated from 400 µL of serum using the QIASYMPHONY automated instrument (QIAGEN, Hilden, Germany). All samples collected before 2019 were retro-transcribed employing the QIAGEN Onestep RT-PCR kit with primers for the HEV ORF2 region as previously described [29]. For samples obtained since 2019, RT-PCR was performed by Super Script IV PCR with random primers (SSIV) (Thermofisher Scientific, Paisley, UK), according to the manufacturer’s instructions. Amplification of cDNA was achieved by a nested polymerase chain reaction protocol (PCR) using TaqGold polymerase and two sets of primers encompassing a 457 bp fragment within the ORF2 gene, as used in the OneStep protocol [29]. Cycling conditions for first and second round PCR were as follows: (1) 95 °C (15 min), (2) 94 °C (30 s), (3) 56 °C (30 s) and (4) 72 °C (45 s); steps 2–4 were repeated 35 times. A final extension was performed at 72 °C for 10 min. ORF2 RT-PCR negative samples were retested with primers against a conserved ORF1 region [30]. RNA was retro-transcribed in the presence of random hexamers and SSIV. cDNA (10 µL) was used as template in the first round PCR with outer primers 1679 and 1680 while nested primers 1681 and 1682 were employed for second-round PCR [30]. Cycling conditions for PCR were: 94 °C, 15 min; 40 cycles at 94 °C for 30 s, 50 °C for 45 s and 72 °C for 1 min; and a final extension at 72 °C for 7 min.

Direct sequencing of both PCR product strands using second round amplification primers was achieved on the ABI PRISM 3100 automated sequences (Applied Biosystems, Forster, CA, USA). Following a preliminary BLAST analysis, the genotype and subtype of each isolate was confirmed by phylogenetic analysis. Sequences were aligned using the MEGA X software package [31] and the maximum–likelihood method based on General Time Reversible (GTR) model +G + I with 500 bootstrap replicates including reference sequences [4]. The phylogenetic tree was visualized with FigTree v. 1.4.4.

Two strains were not included in the phylogenetic analysis since Sanger sequencing only led to resolution of short fragments; these strains were classified as GT3 unsubtyped (Pt21 and Pt34).

## 3. Results

**Figure 1:**
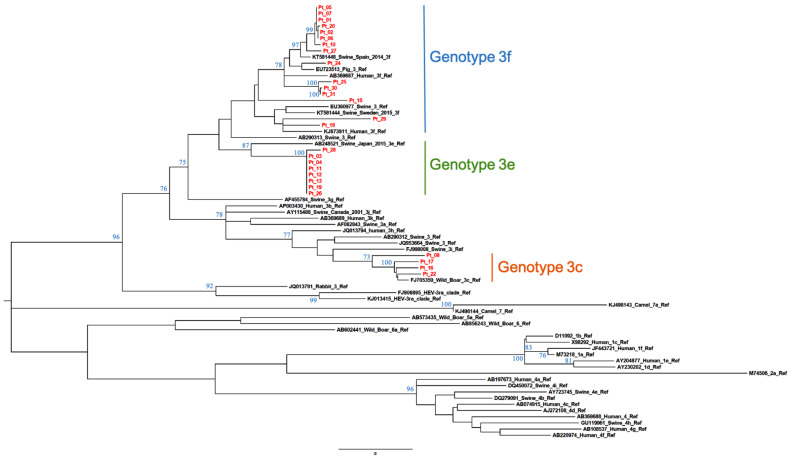
Subtyping of HEV GT3 patient-derived samples by phylogenetic analysis. Twenty-seven ORF2 patient-derived sequence fragments (shown in red) and reference sequences ([4], GenBank accession numbers shown) were used for the construction of a maximum-likelihood tree. Bootstrap values were based on 500 replicates. Bootstrap values > 60% are indicated on respective branches. The scale bar represents nucleotide substitutions per site. GenBank accession number of patient sequences are reported in Table S1 (see Supplemental Material).

**Figure 2:**
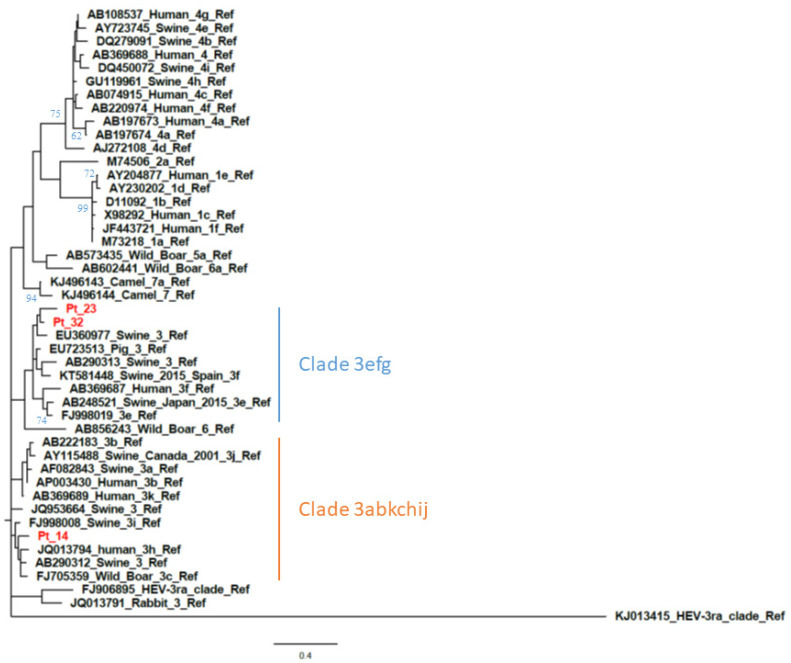
Phylogenetic analysis based on partial open reading frame 1 (ORF1) (125 nt, nt78–202 of the HE-JA04–1911 isolate, GenBank accession number AB248521) sequences from patient-derived samples with a negative ORF2 RT-PCR result. The phylogenetic tree includes the prototype strains as indicated by Smith et al. [4] The maximum-likelihood tree was constructed with a bootstrap of 500 replicates. Bootstrap values > 60% are indicated on respective branches. The scale bar represents nucleotide substitutions per site. Supplementary data on sequence accession number of patients are provided in Table S1 (see Supplemental Material).

The calculated AI was employed to differentiate samples from patients with recent versus resolved HEV infections. Qualitative AIs are listed in Table 1. In group A (*n* = 34), which included samples positive for HEV RNA and anti-HEV IgG/IgM, two were segregated with GT1 while the majority (*n* = 32) were segregated with GT3. The GT3 sequences clustered with the following subtypes or clades: GT3c (*n* = 5), GT3e (*n* = 8), GT3f (*n* = 17) (Figure 1 and Figure 2) while two were GT3-unsubtyped. The majority of group A samples (*n* = 31) had a low AI (<40%); the mean AI was 17.5%.

**Figure 3:**
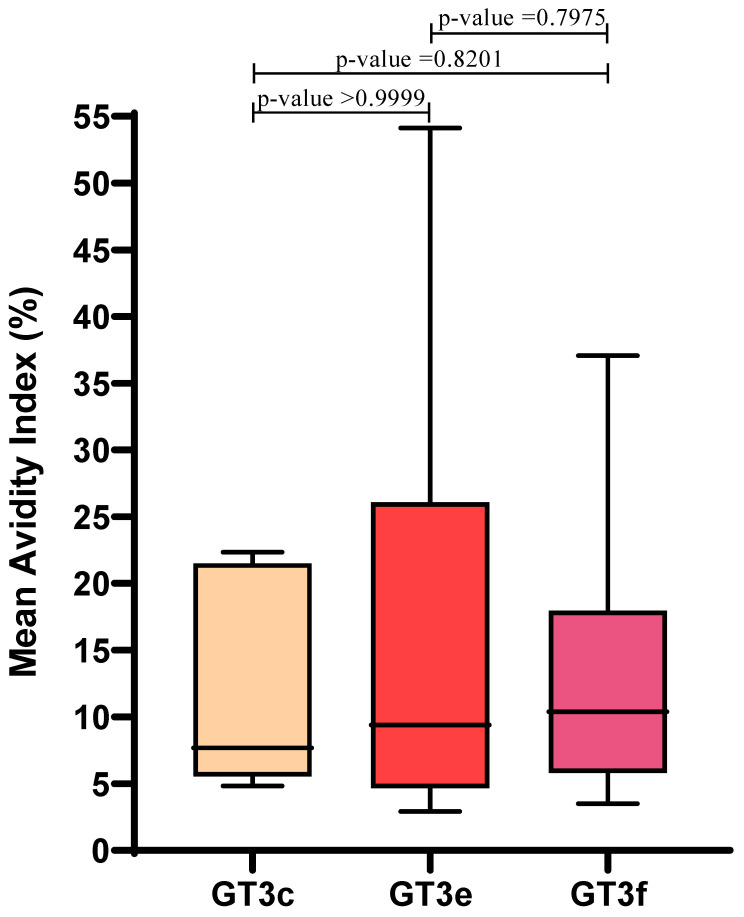
Correlation between HEV GT3 subtypes (GT3c, *n* = 5; GT3e, *n* = 8; GT3f, *n* = 17) and the mean Avidity Index, which was calculated for Group A GT3 patients. Boxes represent the first and third quartiles and the black line represents the median value. *p*-values were calculated using the Mann–Whitney test.

Similar AI values were observed irrespective of GT3 subtype: mean AIs for GT3c, GT3e and GT3f were 12.4 ± 8.4, 16.8 ± 17.4, and 14.1 ± 10.4, respectively. Therefore, in this limited sample set, AI values were not influenced by GT3 subtype (*p* > 0.05) (Figure 3).

For the three patient-derived samples with an AI > 40%, one (Pt33) was a Bengali migrant who had an AI of 44.5% and was hospitalized for a confirmed GT1 acute infection after returning from Bangladesh. Since GT1 is endemic in Bangladesh, it is possible that this equivocal AI value was related to the patient being reinfected; therefore, anti-HEV IgG binding to antigen could be stronger even in the early phase of reinfection. Pt34 (AI = 64.7%) was tested for the presence of anti-HEV IgG and IgM after confirming the absence of other types of viral hepatitis (HAV, HBV and HCV). This patient was still in the late phase of acute infection with detectable HEV RNA, and exhibited a high anti-HEV IgM index (11.5 S/CO) and an O.D. of 3.140, suggesting seroconversion 1–3 months earlier. On subsequent testing a month later, Pt34 was HEV RNA negative. Pt28 (AI = 51.2%) was first shown to be HEV RNA positive two months earlier; this patient was receiving antiviral treatment (sofosbuvir plus ribavirin) for HEV infection.

In Group B (*n* = 16), the mean AI was 73.5% ± 26.8%, and there were three patients with low AI (<40%). One female patient (Pt39) with an AI of 18.6% was pregnant and anti-HEV IgG/IgM positive with transaminase elevation, nausea and pain. All other hepatitis markers were negative, including anti-*Toxoplasma gondii* IgG and IgM. The other two patients (Pt46 and Pt48) had acute hepatitis clinical manifestations although negative for HEV RNA as well as for cytomegalovirus (CMV), EBV, HAV and HBV serological markers. Pt46 (AI = 39.1%) was hospitalized in a different medical centre. The shipped serum sample had not been stored at an optimal temperature; therefore, it is possible that HEV RNA degradation may have occurred before testing. Pt48 (AI = 35.5%) had clinical symptoms indicative of acute infections 6–8 weeks before HEV RNA and antibody testing.

Equivocal values for anti-HEV IgG AI were observed for three patients (Pt38, Pt40 and Pt47) who recovered from hepatitis: they had normalization of their ALT, AST, and total bilirubin values. Speculation that HEV serological testing was performed in the late stages of acute infection could not be confirmed since the timing for onset of symptoms was unknown.

**Figure 4:**
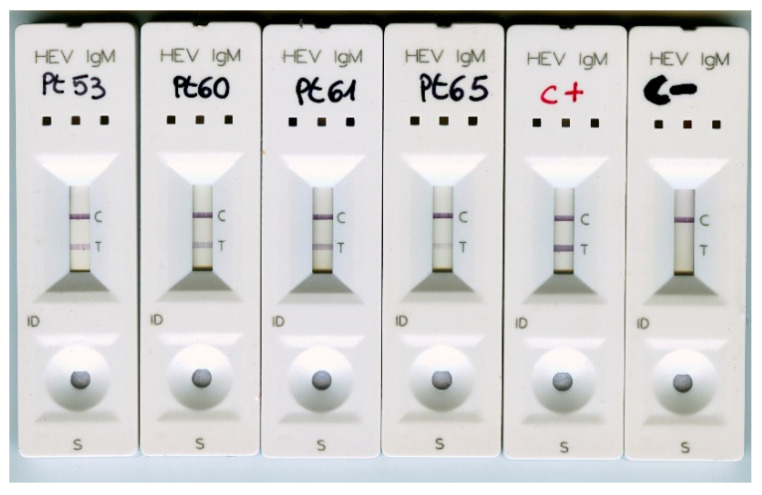
Group C patient samples with persistent (>24 months) anti-HEV IgM positivity were tested using the anti-HEV IgM rapid test. C+, positive control (HEV RNA positive, anti-HEV IgM positive, acute infection); C-, negative control (anti-HEV IgG positive, anti-HEV IgM negative, resolved HEV infection).

Group C represented single or double time-point convalescent patient-derived samples collected 1–27 months after a HEV RNA positive result. In this group, the mean AI was 90.2% ± 20.9%. Three patients were sampled sequentially: two were first sampled during the early stage of acute infection when HEV RNA was positive (Pt3 and Pt12 from Group A) and then approximately 4–5 months later; the third patient (Pt53) was sampled twice during follow-up (Table 1). All Group C patients with persistent (>6 months) anti-HEV IgM exhibited an AI > 70% (Table 2). Patients with a follow-up period > 3 months also showed an AI value > 70%. It is feasible that Pt64 (AI = 28.6%), who was HEV RNA positive one month before the AI determination, were still experiencing a late phase acute infection. Overall, the results demonstrated that low values of AI were indicative of a recent HEV infection, whereas high values of AI suggested a resolved infection. Samples from four patients with persistent anti-HEV IgM for ≥24 months were also tested with the “One site HEV IgM rapid test”. All samples were anti-HEV IgM positive (Figure 4). This further confirmed long-lasting IgM positivity.

Group D patient samples were selected to assess both cross-reactivity and specificity of the AI assay. All HAV-positive samples (*n* = 12), HBV-positive samples (*n* = 1), EBV anti-VCA IgM positive samples (*n* = 10), and anti-CMV IgM positive samples (*n* = 10) produced negative HEV IgG avidity results. Five samples from patients who were anti-HEV IgG positive and IgM negative yielded an AI > 70%. The DIA.PRO HEV IgG avidity test showed no cross-reactivity (samples from Pts 66–104). One patient (Pt71) was viremic and infected with a GT3c strain and, as expected for an early stage acute infection, was negative for both anti-HEV IgG and IgM and had an indeterminate AI. Data analyses indicated that the AI assay sensitivity was 91.2%, the specificity was 100%, and the accuracy was 90.5%.

**Figure 5:**
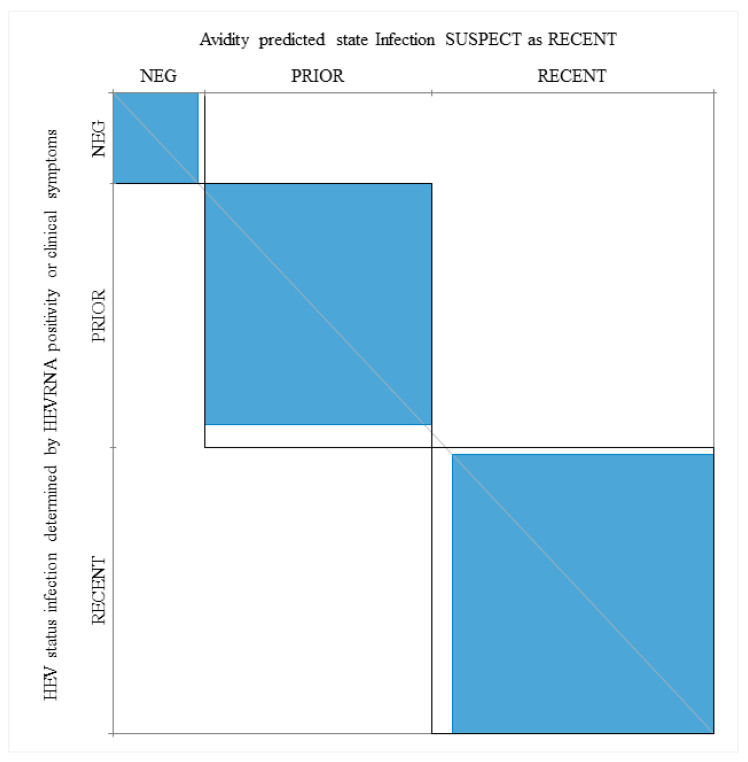
Power of the IgG avidity test in prediction of recent and prior (resolved) infections when considering HEV RNA positivity and clinical manifestations. Neg, patients who are HEV RNA negative and anti-HEV IgM negative. The kappa statistic was employed to measure the agreement between the Avidity Index (AI), HEV RNA, serological markers and clinical symptoms (see Section 3).

When HEV RNA positive patients and patients with clinical manifestations associated with acute infection (P28, Pt38, Pt40, Pt85) were considered as having a recent infection, the agreement between a low AI and recent infection was 97.4%, while the kappa value, as determined by the Wald Test, was 92.3 (Figure 5).

## 4. Discussion

Serological tests such as those detecting anti-HEV IgG and IgM, and molecular tests measuring HEV RNA, are considered the basis for the diagnosis and differentiation between acute and resolved hepatitis E infections [17]. In clinical practice, however, anti-HEV IgM positivity in HEV RNA negative individuals does not provide any clear indication of the infection status, especially since anti-HEV IgM antibodies may persist for months or even years [22,32] following the acute phase of infection. A greater understanding regarding the utility of avidity values for diagnosing HEV disease stage in patients with long-lasting anti-HEV IgM positivity, as well as HEV GT3 subtype effects on avidity test performance is required.

In our study, the performance of the commercially available DIA.PRO assay was evaluated. This assay was employed as an anti-HEV IgG diagnostic test for measuring IgG avidity in HEV RNA positive patients primarily infected with GT3. These GT3 strains were subtyped to assess the impact of subtype on assay proficiency. For Group A patients who were viremic and positive for anti-HEV IgM, the avidity assay sensitivity was 91.2%, confirming results previously described by other authors [27,28]. Assay specificity was 100%, indicating that pathogens such as HAV, which causes acute hepatitis with symptoms similar to those of hepatitis E, were not cross-reactive with the HEV avidity determination. In addition to HAV, HBV, CMV and EBV, active infections did not appear to interfere with the HEV avidity determination; however, further analyses are recommended to confirm these findings.

Only three (<10%) viremic patients (Pt28, Pt33, Pt34) with acute infection showed equivocal AI results.

In contrast to the Bigaillon study [27], we observed no false low-avidity values in sera from immunocompetent patients with a follow-up period >4 months after HEV RNA detection.

However, the relatively low number of samples did not allow any conclusive remarks.

Even Pt63, who was anti-HEV IgM positive four months after being HEV RNA positive, had a high-avidity value (mean AI = 79.5%). Therefore, in our study, a low IgG AI, measured using the DIA.PRO assay, appeared to predict an infection in the early stages (first 3–4 months), while a high IgG AI excluded patients with a primary infection occurring for <4 months. AI results within the equivocal range (>40–70%) are more difficult to interpret. As shown in Table 1, three Group A viremic patients (Pt28, Pt33 and Pt34), diagnosed by clinicians as having an acute HEV infection, and three Group B patients (Pt38, Pt40 and Pt47), with ALT normalization at the time of AI determination, had AIs in the >40–70% range. Additionally, a Group C patient (Pt58) had an equivocal AI two months after being HEV RNA positive. Some equivocal results may be linked to reinfection (Pt33) or a difference in maturation time for IgG avidity (Pt34); however, limitations of available anamnestic data and follow-up serum samples prevented us from confirming these assertions.

No statistically significant differences in AI sensitivity were observed between Group A GT3 viremic patients, irrespective of GT3 subtype (Figure 3). This is notable as it reinforces the value of the avidity test for assessment of acute HEV infections. Recently, Riviero-Barciela proposed this using HEV antigen (HEV-Ag) as a serological marker to discriminate between recent and resolved HEV infections in patients positive for anti-HEV IgM. The authors calculated a predictive positive value of 100% and a negative predictive value of 44% for the HEV-Ag test, resulting in a diagnostic accuracy of 57% [32], and suggested using this test to exclude primary infection in patients with persistent anti-HEV IgM. Of note, HEV-Ag was shown to be weakly sensitive in asymptomatic blood donors [33] and in patients with HEV acute infection [34] where HEV-Ag sensitivity was reported as 40% and 46.7%, respectively. Additionally, Zhao demonstrated that the presence of high IgG concentrations reduced the sensitivity of their HEV-Ag assay [35]. Moreover, Trémaux demonstrated that HEV-Ag assay sensitivity varied depending on GT3 subtype [36]; the lower limit of detection differed by one or two logarithmic units when comparing GT3c versus GT3f. Conversely, our results suggested that AI was not affected by the GT3 subtype during the assessment of acute infections and we were able to identify recent infections in HEV RNA-negative patients with clinical symptoms.

## 5. Conclusions

Results described in this study support the application of the anti-HEV IgG avidity ELISA test for distinguishing between recently acquired and resolved infections, irrespective of anti-HEV IgM positivity status. Although the sample size was limited, the results suggested that the avidity test performance appeared to be reliable for acute infection assessments of HEV GT3 subtypes, which is in contrast to observations with the reported HEV-Ag assay [36]. For avidity tests yielding equivocal AI values, potentially caused by the variability in timing for IgG maturation, recent HEV infections should be confirmed by HEV RNA detection.

## Figures and Tables

**Table 1:** Patient serological status, HEV RNA results and avidity index calculations.

Sample Identification	Sex	Date of Sampling	HEV RT-PCR	HEV Genotype	Mean Avidity Index (±SD)	HEV-IgG	HEV-IgM	Follow-Up ^§^	Other
**Group A**									
Pt1	M	31/10/2019	+	3f	4.5 (1.1)	+	+		
Pt2	M	27/01/2020	+	3f	9.0 (1.7)	+	+		
Pt3	F	13/08/2019	+	3e	10.9 (2.2)	+	+		
Pt4	F	30/09/2019	+	3e	28.0 (3.0)	+	+		
Pt5	M	18/10/2019	+	3f	7.1 (3.4)	+	+		
Pt6	M	08/10/2019	+	3f	6.5 (1.0)	+	+		
Pt7	M	04/11/2019	+	3f	14.9 (1.5)	+	+		
Pt8	M	27/01/2020	+	3c	20.7 (3.8)	+	+		
Pt9	M	31/05/2017	+	1	29.2 (4.0)	+	+		
Pt10	M	06/12/2019	+	3f	9.0 (1.6)	+	+		
Pt11	M	22/07/2019	+	3e	4.4 (1.1)	+	+		
Pt12	M	27/08/2019	+	3e	5.5 (0.6)	+	+		
Pt13	M	10/09/2019	+	3e	7.9 (0.3)	+	+		
Pt14	M	26/01/2018	+	3c	4.8 (0.7)	+	+		
Pt15	M	17/03/2018	+	3f	14.9 (12.4)	+	+		
Pt16	M	08/05/2020	+	3c	7.7 (1.0)	+	+		
Pt17	M	17/01/2019	+	3c	6.3 (2.5)	+	+		
Pt18	M	18/01/2019	+	3f	19.6 (2.6)	+	+		
Pt19	M	24/08/2019	+	3e	20.2 (3.6)	+	+		
Pt20	M	18/10/2018	+	3f	3.5 (1.1)	+	+		
Pt21	M	03/12/2019	+	3	25.3 (1.3)	+	+		
Pt22	M	21/01/2016	+	3c	22.3 (0.8)	+	+		
Pt23	M	03/03/2018	+	3f	28.3 (0.8)	+	+		
Pt24	M	27/06/2020	+	3f	37.1 (2.4)	+	+		
Pt25	M	03/02/2016	+	3f	16.3 (0.5)	+	+		
Pt26	M	24/08/2019	+	3e	2.9 (0,3)	+	+		
Pt27	M	25/05/2017	+	3f	35.5 (1.2)	+	+		
Pt28	M	01/03/2016	+	3e	54.1 (6.2)	+	+		
Pt29	M	30/03/2018	+	3f	8.0 (1.0)	+	+		
Pt30	F	05/05/2018	+	3f	5.1 (0.6)	+	+		
Pt31	M	31/05/2018	+	3f	11.9 (0.4)	+	+		
Pt32	M	14/07/2018	+	3f	6.0 (0.2)	+	+		
Pt33	M	13/06/2019	+	1	44.5 (0.7)	+	+		
Pt34	F	23/06/2020	+	3	63.4 (1.2)	+	+		
**Group B**									
Pt35	M	17/10/2018	−	n.d.	83.7 (0.8)	+	+		
Pt36	M	16/11/2019	−	n.d.	95.0 (0.9)	+	+		
Pt37	F	17/12/2019	−	n.d.	71.2 (2.7)	+	+		
Pt38	M	31/08/2019	−	n.d.	49.1 (1.3)	+	+		
Pt39	F	25/10/2019	−	n.d.	18.1 (0.3)	+	+		
Pt40	F	26/10/2019	−	n.d.	59.2 (0.4)	+	+		
Pt41	M	28/03/2018	−	n.d.	87.6 (0.9)	+	+		
Pt42	M	07/03/2018	−	n.d.	79.8 (0.4)	+	+		
Pt43	M	19/06/2019	−	n.d.	92.5 (0.7)	+	+		
Pt44	M	30/08/2019	−	n.d.	100.0 (0.5)	+	+		
Pt45	F	21/01/2019	−	n.d.	100.0 (0.0)	+	+		
Pt46	M	22/01/2019	−	n.d.	38.8 (1.1)	+	+		
Pt47	F	26/10/2019	−	n.d.	64.6 (1.0)	+	+		
Pt48	M	30/10/2019	−	n.d.	34.7 (20.1)	+	+		
Pt49	M	19/10/2016	−	n.d.	100.0 (0.0)	+	+		
Pt50	M	13/11/2019	-	n.d.	100.0 (0.0)	+	+		
**Group C**									
Pt51	M	20/02/2016	−	n.d.	98.4 (0.4)	+	+	8 months	
Pt52 *	M	22/02/2020	−	n.d.	100.0 (0.0)	+	+	5 months	
Pt53 ***	M	12/02/2018	−	n.d.	98.9 (0.3)	+	+	24 months	
Pt54	M	30/08/2019	−	n.d.	99.4 (1.1)	+	+	14 months	
Pt55	M	14/10/2018	−	n.d.	100.0 (0.0)	+	+	7 months	
Pt56	M	23/04/2018	−	n.d.	100.0 (0.0)	+	+	7 months	
Pt57	F	28/10/2018	−	n.d.	99.8 (0.2)	+	+	14 months	
Pt58	M	18/06/2018	−	n.d.	55.4 (0.7)	+	+	2 months	
Pt59	M	04/06/2019	−	n.d.	100,0 (0.1)	+	+	15 months	
Pt60 ***	M	19/05/2018	−	n.d.	99.2 (0.7)	+	+	27 months	
Pt61	M	23/03/2020	−	n.d.	100.0 (0.0)	+	+	24 months	
Pt62	M	15/03/2018	−	n.d.	96.3 (0.8)	+	+	12 months	
Pt63 **	F	04/12/2019	−	n.d.	79.5 (1.3)	+	+	4 months	
Pt64	M	16/02/2016	−	n.d.	28.5 (1.3)	+	+	1 month	
Pt65	M	19/07/2018	-	n.d.	95.8 (1.4)	+	+	25 months	
**Group D**									
Pt66	M	23/10/2016	−	n.d.	100.0 (0.0)	+	−		
Pt67	F	24/11/2015	−	n.d.	100.0 (0.0)	+	−		
Pt68	M	05/12/2015	−	n.d.	72.2 (5.6)	+	−		
Pt69	M	11/05/2020	−	n.d.	93.8 (2.5)	+	−		
Pt70	M	11/05/2020	−	n.d.	98.6 (0.6)	+	−		
Pt71	F	17/06/2020	+	3c	n.d.	−	−		
Pt72	M	03/06/2019	−	n.d.	100.0 (0.0)	+	+		anti_HBc IgM +, HBV DNA +
Pt73–Pt84	M = 11/F = 1	02/03/2017–15/04/2020	−	n.d.	n.d.	−	−		HAV RNA +
Pt85–Pt94	M = 6/F = 4	23/02/2016–26/09/2020	−	n.d.	n.d.	−	−		VCA_IgM +, EBV DNA +
Pt95–Pt104	M = 0/F = 10	05/06/2020–11/06/2020	-	n.d.	n.d.	-	-		anti_CMV-IgM, CMV DNA +

n.d., not determined; +, positive; −, negative; CMV, Cytomegalovirus; ^§^ Follow-up visit in months after HEV RNA was detected; * Follow up of Pt12; ** Follow up of Pt3; *** These samples represent two different follow-up visits for the same patient.

**Table 2:** Group C HEV IgG avidity results categorized by the estimated time since HEV RNA detection.

Time of AI Detection	Low AI	Equivocal AI	High AI
≥5 months	0	0	11
>2 month, <5 months	0	0	2
>1 month, ≤2 months	0	1	0
≤1 month	1	0	0

AI, Avidity Index; Low AI: 1–40%; Equivocal AI: >40–70%; High AI: >70%.

## Supplementary Materials

The following are available online at https://www.mdpi.com/1999-4915/13/2/236/s1, Table S1: GenBank accession numbers for sequences included in phylogenetic trees targeting ORF1 or ORF2.
