# Supplementary material for: Anti-HEV IgG Avidity Testing: Utility for Diagnosing Acute and Resolved Genotype 3 Infections
Source: Viruses. 2021 Feb 3;13(2):236. doi: 10.3390/v13020236 (PMC7913725; doi:10.3390/v13020236)
Supplement: Supplementary file 1 [file viruses-13-00236-s001.pdf]

**Table S1.** GenBank accession numbers for sequences included in phylogenetic trees targeting ORF1 or ORF2.

| | GenBank Accession Number | |
|---|---|---|
| **Code Patient** | **ORF1** | **ORF2** |
| Pt-01 | - | MT769324 |
| Pt-02 | - | MW013052 |
| Pt-03 | - | MT763867 |
| Pt-04 | - | MT769323 |
| Pt-05 | - | MN737483 |
| Pt-06 | - | MN737483 |
| Pt-07 | - | MT69325 |
| Pt-08 | - | MT990345 |
| Pt-010 | - | MT263983 |
| Pt-011 | - | MT763865 |
| Pt-012 | - | MT769321 |
| Pt-013 | - | MT769322 |
| Pt-015 | - | MN444853 |
| Pt-016 | - | MW013051 |
| Pt-017 | - | MN537875 |
| Pt-018 | - | MN537876 |
| Pt-019 | - | MN497623 |
| Pt-020 | - | MN737484 |
| Pt-022 | - | MN444839 |
| Pt-024 | - | MW013051 |
| Pt-025 | - | MN444840 |
| Pt-026 | - | MT763866 |
| Pt-027 | - | MN444828 |
| Pt-028 | - | MN444841 |
| Pt-029 | - | MT86652 |
| Pt-030 | - | MN444852 |
| Pt-031 | - | MN444849 |
| Pt-014 | MT763859 | - |
| Pt-023 | MT763861 | - |
| Pt-032 | MT763862 | - |
